# Perceptions of childhood undernutrition among rural households on the Kenyan coast – a qualitative study

**DOI:** 10.1186/s12889-016-3157-z

**Published:** 2016-08-02

**Authors:** Kelly W. Muraya, Caroline Jones, James A. Berkley, Sassy Molyneux

**Affiliations:** 1 KEMRI-Wellcome Trust Research Programme, P.O. Box 230-80108, Kilifi, Kenya; 2 Centre for Tropical Medicine & Global Health, Nuffield Department of Medicine, University of Oxford, Old Road Campus, Headington, Oxford, OX3 7BN UK

**Keywords:** Child, Illness perception, Nutrition, Gender, Rural households

## Abstract

**Background:**

Nutrition plays an important role in child survival and development. Treatment action in the management of child health and nutrition is influenced by perceptions of illness, and gender plays an important role. However, little is known about if and how moderate undernutrition is recognised among lay populations, or how local social norms and intra-household dynamics affect decisions to seek biomedical assistance for nutritional concerns. In this paper we describe how childhood nutritional problems are recognised and understood within rural households. We demonstrate how context influences local constructs of ‘normal’, and suggest the centrality of gender in the management of child health and nutrition in our research context.

**Methods:**

This qualitative study was undertaken in Kilifi County on the Kenyan Coast. A set of 15 households whose children were engaged in a community-based nutrition intervention were followed up over a period of twelve months. Over a total of 54 household visits, group and individual in-depth interviews were conducted with a range of respondents, supplemented by non-participant observations. Eight in-depth interviews with community representatives were also conducted.

**Results:**

Local taxonomies of childhood undernutrition were found to overlap with, but differ from, biomedical categories. In particular, moderate undernutrition was generally not recognised as a health problem requiring treatment action, but rather as routine and manageable, typically seasonal, weight-loss. Where symptoms were considered more serious and requiring remedial action, household management strategies were typically based on perceived aetiology of the illness. Additionally, gender emerged as a potentially central theme in childhood nutrition problems and related management. Women reported that they have primary responsibility for ensuring children’s good health and nutritional status, and that they are often held accountable when their children are of sub-optimal health.

**Conclusion:**

Perceptions of child nutrition and illness and gendered roles within households influence treatment action, and engagement with nutrition interventions. Community-based nutrition interventions must recognise these complex realities.

## Background

Nutrition affects health throughout the human life-course and plays a critical role in cognitive, motor and social development, particularly in early childhood [1, 2]. Both over- and undernutrition increase the risk of disease and early death, with young children being most vulnerable [3–5]. Overall, 3.1 million childhood deaths annually have been attributed to undernutrition [2]. It is estimated that more than 80 % of the global burden of child undernutrition occurs in 24 countries [5], the majority of which are in Asia and sub-Saharan Africa [1, 2, 5].

Biomedicine broadly categorises undernutrition as: wasting, stunting, and underweight [5–7], and these are further classified into either moderate or severe undernutrition. The World Health Organisation (WHO) classifies these measures using calculated Z scores, indicating the number of standard deviations away from the median of an international healthy reference population: <-2z indicating moderate and < -3z indicating severe categories [7]. The importance of moderate degrees of undernutrition to child health is becoming better recognized among biomedical and international development communities; and there has been increased implementation of community-based nutrition interventions aimed at addressing this [8–11].

Wasting, defined as low weight for height, is regarded as an indicator of acute malnutrition. It suggests recent inadequate food intake or acute infection resulting in anorexia and malabsorption of nutrients [5]. Stunting, defined as low height for age, is regarded as an indicator of the collective effects of chronic undernutrition and illness [5]. Besides an increased risk of death, stunting is associated with impaired long-term developmental and reduced economic capacity [5, 6, 12]. Its prevention is typically a target of development programmes and no effective treatment for established stunting is in use. Low weight for age (underweight) was previously the main anthropometric indicator for children and is still widely used for growth monitoring [2, 5] and in emergency situations [13]. It is, however, now recognized that weight for age gives the least clear picture of child nutritional status. For example, a tall wasted child can have normal weight for age. Hence, the shift to other measures including the mid-upper arm circumference (MUAC) [2, 13]. The presence of oedema - that is, fluid build-up in the body tissues or cavities resulting in swelling of affected areas - is also used to define kwashiorkor. Kwashiorkor is a form of severe acute malnutrition as a result of protein deficiency in the diet [14]. Treatment is generally integrated within clinical services and its aims are to prevent death and achieve nutritional recovery.

Recognition that a child is undernourished underpins efforts to improve their nutritional status. However, understanding of undernutrition, its causes and its identification vary across cultures, medical systems and between lay and health professional models. Lay explanations may not align with those of biomedicine. Examples of alternative explanations cited for symptoms classified as undernutrition by biomedicine include: breaches of cultural restrictions that govern relationships particularly sexual relations; disregard of taboos and other ancestral or religious responsibilities; breastfeeding by a pregnant mother – for example leading to the swollen belly of a child suffering from kwashiorkor; practising sorcery and witchcraft; or the belief that some children are inherently prone to nutritional disorders [15–23]. However, little is known about if or how moderate undernutrition is recognised in vulnerable communities, or how local social norms and intra-household dynamics affect decisions to seek biomedical assistance for nutritional concerns.

Treatment-seeking behaviour for illness including nutrition-related disorders is often a complex process influenced by multiple factors [24–28]. In particular, household decision-making processes and differential access to resources based on gender can have an important impact on child health, nutrition and treatment-seeking behaviour [29–32]. In many rural African settings, gender - its intersectionality with other social axes - and family relations; strongly influence treatment decision-making processes for child illness [17–19, 27, 28, 33]. Women are often held responsible for the health of their children, but many household members and other social network members can be involved in treatment seeking actions [17, 18, 23, 26, 33–35]. Whether or not mothers make independent decisions in relation to child illnesses is determined by a range of inter-related factors including: the nature and perceived severity of illness; who is perceived to own the child; what is perceived to have caused the illness; and intra-household roles and relations [27, 28, 33]. This suggests that understanding the dynamics of perceptions of illness, gender intra-household relations, and how these interact with recognition of child undernutrition, subsequent treatment, and interactions with nutrition interventions is crucial to addressing the malnutrition situation.

We aimed to understand how childhood nutrition problems are recognised and understood in households, and the treatment that is sought for children who are perceived to be unhealthy; demonstrating the importance of context in local constructs of ‘normal’ and the potential centrality of gender in the management of child health and nutrition in the study community. We explored the interactions between intra-household gender relations and community-based nutrition interventions, and a range of household factors that may contribute to nutritional status and influence engagement with nutrition interventions.

## Methods

### Study site

This study was undertaken in Kilifi County at the Coast of Kenya. The majority of residents are Giriama, a sub-group of the Mijikenda ethnic group. The county has low literacy levels particularly amongst females, and very high levels of poverty [36, 37]. The primary economic activity is small scale farming which is heavily dependent on seasonal rainfall, rendering the area prone to frequent food insecurity necessitating emergency food relief operations [36, 38]. Kilifi has high rates of child undernutrition. A survey undertaken in the area in 2011 placed global acute malnutrition at 4.0 % and severe acute malnutrition at 0.7 % [38]. The prevalence of stunting was extremely high at 48.8 and 19.6 % for severe stunting, while global underweight was 21.3 % with severe underweight at 5 % [38].

### Selection of study households and data collection

In this qualitative study, fifteen households whose children were engaged in the Supplementary Feeding Programme (SFP) – a community-based nutrition intervention targeting children aged 6-59 months with moderate acute malnutrition; were followed up over 12 months. All the households approached for this study agreed to participate. In total, there were 24 children involved in the SFP within the selected homes. The fifteen households were purposively selected to reflect the diversity of homes involved in the intervention. Criteria for household selection were guided by factors cited in literature as impacting on household relations and health decision-making dynamics [16–19, 21, 23, 27, 39]. In particular, household structure and headship are central to family dynamics and were key selection criteria. Additional factors considered in selecting the households included: level of engagement with the SFP; experience of varying intervention products such as flour and ready-to-use supplementary foods (Plumpy-sup®); residency of the target child’s father; and formal education of the target child’s primary carer.

Interviews were conducted with a range of respondents within the homes and typically included: parents and primary carers of children enrolled in the intervention; grandparents, aunts and uncles of the target child; and co-wives of the target child’s mother. The selected households were visited four to six times over the 12-month period, totalling 54 household visits. A range of topics relating to child and household feeding practices, child health, treatment actions during child ill health and experiences of community-based nutrition interventions were covered during the household visits. This iterative and longitudinal approach facilitated greater understanding of the topics under investigation, and allowed for variations in household feeding practices over time and by season to be documented. Nevertheless, due to poor rainfall levels during the study period (and in preceding years), the entire data collection period was generally dry with high food scarcity. Eight in-depth interviews with both male and female community representatives were also undertaken to supplement the information obtained from households. Interviews were primarily conducted in the local Giriama language and then translated into English.

Two field staff - one male and one female - who themselves came from the local community, also provided valuable contextual information on community norms, practices and beliefs. Added advantages of working with local field staff were support in gaining entry into homes, and in establishing trust and rapport with household members. This was essential given the extensive engagement with households over a prolonged period of time. Having both a male and female fieldworker also allowed for gender sensitivities to be responded to in locally appropriate ways. In the initial visits, the primary author visited each home with both field staff to test for any apparent discomfort in households of interacting with either fieldworker. Given no apparent gender-related concerns, each fieldworker worked with pragmatically allocated households, with the lead author present in all household interactions.

### Data analysis

The data from this work were managed using Nvivo 10 and analysed using a framework analysis approach [40]. After immersion in the data and extensive familiarization with the interview transcripts and field notes, an initial content analysis was undertaken to categorize recurrent themes. The initial themes and sub-themes were based on the study objectives as well as emerging from the data. These themes then formed the coding scheme. The coding process involved splitting and rearranging the data from all the households and respondents according to thematic content. Coding charts were developed to enable comparisons between and within households, as well as between the different types of respondents. The analysis process also involved exploring relationships and associations between concepts and linking the findings to wider literature and theory.

The entire research team was involved in the data analysis process. This allowed for extensive consultation and discussion on emerging issues and development of the coding scheme and charts. Furthermore, given the iterative nature of the study, preliminary findings were discussed with participants in later household visits which enabled discussion of the researchers’ interpretation of the findings. In this way, the respondents became a part of the data analysis process and contributed to further enrichment of the data.

### Ethics & reflexivity

Ethics approval was obtained from the Kenya Medical Research Institute (KEMRI) Scientific and Ethics Review Unit (SSC No. 2099). At initial visits to each household, detailed verbal consent was sought first from the household head or their representative, and then from all adults potentially involved in the study. This process included asking for specific consent to audio-record the interviews, and question and answer sessions to raise any concerns. Written informed consent was obtained from all members of the home who participated in the group and individual interviews. Where a respondent could not read or write, a literate independent witness of their choice was present during the consenting process. In all subsequent household visits, a rapid study reminder was given, and verbal consent to continue obtained. A similar consenting process was followed for the in-depth interviews with community representatives. Given the substantial amount of time spent in each home during the household visits, a modest food package was given to each home at every visit. This was to compensate for work lost for instance from farming to participate in the interviews, and also to thank respondents for their time. For participant anonymity and confidentiality, all identifiers have been replaced with pseudo-names or numeric codes.

Throughout the study, the primary author maintained a reflective diary documenting her thoughts on the research process and the influence of her positionality on the data collection and interpretation of the findings. Her position as a Kenyan but non-indigenous researcher from a well-resourced health research centre, interacting with community members from a poorer socio-economic background, necessarily impacted on the researcher-participant dynamics. Potential adverse perceptions or interactions were minimised through the regular engagement with the participants, which helped to establish trust, rapport and mutual respect. The study team also held frequent de-brief meetings where any arising dilemmas and issues were discussed, including reflecting on the role and positionality of all team members in the data collection and analysis process.

## Results

The children enrolled in the SFP lived in diverse households, often with complex and highly dynamic family relationships. Seven of the 15 households were polygamous and 8 non-polygamous. In both these categories, some households were male-headed and some female-headed. Also, some homes comprised of extended families consisting of multiple nuclear families within one compound, while others were nuclear families of varying sizes. The focus of the study was the household but it became clear across the 12 months of the study that women played the major role in day to day decisions about a child’s food intake, and it was the women who were most keen to engage in discussions about child health. Men were often away from households when the study team visited, either because they lived and worked elsewhere or were out for the day. When male members of the household were around, they generally preferred not to join discussions on child health and nutrition as these were considered to be primarily in the “women’s sphere”.

### Notions of child undernutrition overlap but differ from biomedical categories

There was no local direct equivalent of the term ‘undernutrition’ in the Giriama language. As such, in our discussions we commonly used the Swahili equivalent (*ukosefu wa lishe bora –* which directly translates to ‘lack of proper nutrition’), as the majority of respondents were conversant in both Swahili and Giriama. The terminology used to describe nutrition-related disorders in this community overlapped with, but differed from, biomedical terminology.

**Fig. 1:**
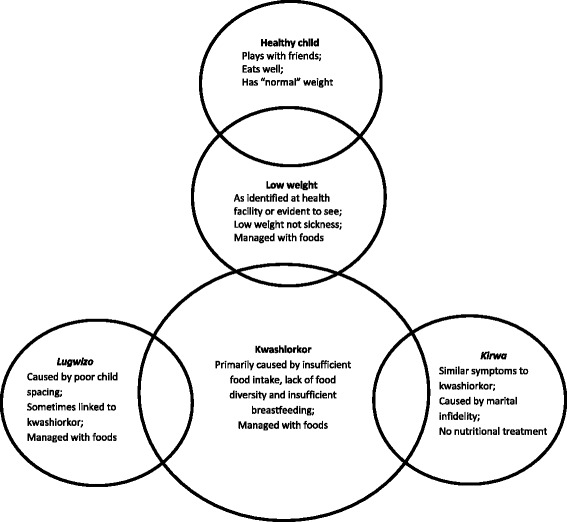
Overlap between child healthiness, low weight and health problems associated with undernutrition

In this community, descriptions were fluid in relation to their children’s health or growth, rather than the rigid categories defined for biomedical diagnosis and treatment. Within the community, whilst there was agreement in recognising a healthy child, there was less clarity about the health of a child who was perceived as underweight. Additionally, although there was consensus that some children were definitely undernourished, a range of terminologies with overlapping descriptions were used. Table 1 summarizes perceived causes, symptoms and management of illnesses associated with undernutrition. Figure 1 is a diagrammatic representation of the fluidity in terms and categorisations, illustrating the move from the perceived healthy child to one with sub-optimal health; and the overlaps between the various illnesses associated with child undernutrition in this setting .

**Table 1:** Summary of perceived causes, symptoms, and management of child health problems associated with undernutrition

Illness	Perceived cause	Described symptoms	Management
Kwashiorkor	Primary causes:	• Swollen/protruding belly • Swollen eyes and cheeks • Thin or emaciated limbs • Reddish/brown thinning hair • Wrinkled or dry skin • Peeling skin • Weak and shrivelled • General poor health	• Primarily food-based • Buy medicine • Attend health facility
	• Food deficiency/lack of food diversity • Insufficient or early cessation of breastfeeding		
	Other causes:		
	• Poor child spacing • Child’s inherent vulnerability • Intestinal worms • Blood deficiency		
*Kirwa*	Either:	• Thin and emaciated • Dry and wrinkled skin • Weak and of general poor health • Folds or crosses their arms • Holds their chin (*kushika tama*)	Traditional treatment
	• Sexual transgression by parent during mother’s pregnancy or • Food deficiency		
*Lugwizo*	Poor child spacing thus:	• Swollen cheeks • Reddish hair • Wrinkled skin • General poor health • Inability to walk • Constant diarrhoea	Food-based
	• Early cessation of breastfeeding or • Breastfeeding from pregnant mother

### Low weight

Low weight (as measured by health workers or perceived by community respondents) frequently emerged as a theme in discussions of child nutrition, although it was not necessarily viewed as indicative, or a category of undernutrition. For most respondents, a child with low weight appeared moderately thin or felt light in weight for their age, but was not something of great concern to carers in comparison to other health issues discussed. In fact, despite all children in this study being categorized at the local health facility as having moderate acute malnutrition, all the carers reported that they did not consider their children to be undernourished; rather as “only having low weight”. Being underweight was also clearly distinguished from illness, with a number of respondents expressing this distinction in discussions about their children’s enrolment into the Supplementary Feeding Programme.

*“I didn’t think there was any problem with the twins. I just took them to the Dispensary for routine clinic. They were weighed and I was told that they have low weight and should therefore take the [Plumpy-sup®]. But it’s just that the kilos were low, there wasn’t any illness that they [health workers] were trying to treat…”* (Karisa homestead_visit 4, index child’s mother)

This is not to suggest that low weight was viewed as entirely unproblematic. Many respondents recognised that low weight was a likely indicator of poor child health. However, low weight was often considered manageable relative to other household priorities and concerns, and to occur with such frequency, particularly during the dry season, that there was a certain normalcy about it. The primary reasons cited for low weight were: insufficient food intake and particularly lack of nutritious foods, as well as lack of food diversity. Other reasons cited for low weight included: illness, for instance the child may get a fever and have reduced appetite; insufficient or early cessation of breastfeeding; and poor child spacing.

### Kwashiorkor

In this community, kwashiorkor was the term that most commonly emerged in discussions of child undernutrition, and was indicated as the term that would likely be used to describe a child who was of sub-optimal nutritional status. Quite often respondents used ‘kwashiorkor’ as a generic term to indicate all symptoms regarded as child undernutrition, including those that might otherwise be used to describe severe wasting from a biomedical perspective. The range of symptoms typically associated with kwashiorkor, and by extension child undernutrition in this setting included: a protruding belly; discolouration and thinning of the hair; swollen eyes and cheeks; patchy, wrinkled or dry skin; peeling skin; thin or emaciated limbs; and general weakness and poor health. In contrast to low weight, children exhibiting such symptoms were viewed as having a serious nutritional problem that would be considered an illness requiring remedial action.

There were a range of explanations given for kwashiorkor, many of them similar to the reported causes of low weight. The two most commonly cited causes were general insufficient food intake or lack of food diversity; and insufficient or early cessation of breastfeeding. Other lesser-cited reasons for kwashiorkor included: poor child spacing; “nature” or the child’s inherent vulnerability where the child is seen as born predisposed to getting kwashiorkor regardless of the care and nutrition they receive; intestinal worms; and blood deficiency.

### Kirwa

Another illness that was regularly discussed in relation to child malnutrition was *kirwa*. Traditionally, *kirwa* was an illness thought to occur as a result of breach in cultural prescriptions relating to sexual relations. Specifically, it was believed to occur due to sexual infidelity perpetrated by either parent whilst the mother was pregnant. When this happened, it was said that the unborn child “had been overtaken” (“*mtoto amepitwa*”), and that the child’s strength was taken away by the unfaithful parent to their extramarital partner.

*“You know according to our culture, when the mother is pregnant, the man should not go outside of the marriage. He should not have sex with any other woman until the mother gives birth, or that mother should not go anywhere [have extramarital affairs] until she delivers. [If this happens] the child will be born with poor health…the father will have taken away the child’s strength to wherever he goes…that is called kirwa.”* (CRF001, female community health worker)

*“It’s like my sister-in-law; I hear things went like this like this, but you know these things are never discussed publicly. You have a husband but you’re not satisfied so you decide to go outside of the marriage. When you do that, it’s like you’ve gone past the unborn child…”* (CRF003, female community health worker)

*Kirwa* was, however, often discussed in the context of kwashiorkor. Similar symptoms described for the two illnesses included the child being thin, weak, emaciated and having dry or wrinkled skin. One key difference was that for the case of *kirwa*, children were always described as folding or crossing their arms and legs. Additionally, *kirwa* tended to be discussed as affecting new born children whereas kwashiorkor affected children aged at least one year and above. There were two broad categories of respondents in this regard: those who described *kirwa* and kwashiorkor as two entirely different illnesses with differing causes; and those who said that *kirwa* and kwashiorkor were one and the same illness. The former maintained that kwashiorkor was a form of, or occurred as a result of, nutritional deficiency. *Kirwa* on the other hand happened when parents failed to abide by cultural restrictions that dictated sexual behaviour during pregnancy. Conversely, those who viewed the two illnesses as similar usually framed it in the context of traditional beliefs versus current knowledge. In other words, kwashiorkor had always existed with a different name and was simply renamed or termed differently with the advent of biomedicine in the area. Thus, what was previously thought to be *kirwa* was in reality just a manifestation of poor child nutrition.

*“Nowadays…people are westernised and have abandoned our cultural ways. The kirwa she [other respondent] is talking about…that disease is the one they are now calling kwashiorkor which needs special foods to replenish the energy in the child’s body, but back then it was called kirwa.”* (Furaha household, target child’s grandfather)

### Lugwizo

Another illness that was commonly referred to in relation to child undernutrition was *lugwizo*. This was also occasionally described as being the same as or a precursor to kwashiorkor. The majority of the symptoms described for *lugwizo* were similar to those of kwashiorkor, except that in the former the child was also unable to walk and would often have diarrhoea. *Lugwizo* was essentially defined as occurring as a result of poor child spacing. In the study community, there are implicit cultural constraints that dictate appropriate child spacing, and any breach of these restrictions can be seen to result in poor health of the most recently born child. Respondents commonly stated that *lugwizo* occurs when a mother falls pregnant before her previously born child is able to walk, or when a mother falls pregnant “too quickly”.

Breastfeeding (or lack thereof) dominated discussions of *lugwizo* and was closely linked with causation of the illness. In this community, a mother was expected to cease breastfeeding immediately she discovered that she was pregnant. It was often said that when the mother falls pregnant her breast milk turns into water *(“maziwa yanatumbuka”*) and is no longer suitable for consumption. Consequently, if a breastfeeding child consumes this milk, they suffer adverse health consequences. The early cessation of breastfeeding due to pregnancy was itself also considered to have implications for the child’s health as was noted in discussions around causes of kwashiorkor and low weight.

In addition to these illnesses and their perceived causes, evil spirits (*mapepo*) were occasionally blamed for child health problems associated with undernutrition. Evil spirits were sometimes directly blamed for causing the problem, for instance a child might be considered to be emaciated as a result of magical powers. Other times spirits were believed to be indirectly responsible for the problem. For example, the mother’s breast milk was somehow affected, and therefore the family must visit a healer to get charms to wade off evil spirits before a new-born child starts to breastfeed.

### Management of child health problems associated with undernutrition

Aside from *kirwa*, management of all the other illnesses associated with undernutrition was cited as mainly food-based. Provision of highly nutritious and diverse foods to the ailing child was viewed as the initial and foremost treatment action. It was only when the “food therapy” failed, particularly where symptoms were perceived as more serious, that some carers stated they would consider seeking further treatment at health facilities. In contrast, treatment of *kirwa* was entirely herbs and traditional healer-based. Where there was suspected infidelity, the mother was advised to consume certain herbs and traditional medicines pre-birth that would ensure that the child was born healthy. Alternatively, certain rituals had to be performed on the afflicted child by a traditional healer. Sometimes, the healer was consulted to give diagnosis or verify if the child was suffering from either *kirwa* or kwashiorkor, which would then determine subsequent treatment action.

Furthermore, though the majority of respondents cited food as the cure for low weight, there was a particular herb “*ambari*” that was repeatedly mentioned with regards to low weight. *Ambari* was generally described as a herb or herbal drink that was used to help add weight or to “make children fat”. Some respondents stated that *ambari* could be used by both adults and children who wanted to “be fat” and look healthy, whereas others referred to it as mostly being used for children who appeared small and unhealthy. Most of the respondents however spoke of *ambari* as something that they had heard of, or they would cite an example of a particular person within the community who was rumoured to have used it, and stated that they knew little about it themselves. Frequently, use of *ambari* was discussed as a traditional practice that had now ceased except in particular pockets of the community.

### Gender in the causes and management of illness

In the study community, women were broadly viewed as almost entirely responsible for ensuring good child health and nutrition, and playing the primary role in the prevention and management of child health problems associated with undernutrition.

*“For an [underweight] child to add weight and have good health, depends on the mother’s hard work. You should struggle to give the child a variety of foods all the time.”* (Safari homestead_visit 3, index child’s mother)

This was despite mothers’ - especially younger mothers’ - often limited control of household resources, which sometimes restricted their ability to provide nutritious diets for their children. The majority of husbands and fathers lived and worked elsewhere, sending in remittances to the family home every month or so, with important implications for family dynamics and hierarchies. For example young women’s money was not always sent directly to the woman, but to an older female or male in the household, influencing her ability to make independent decisions regarding her children’s health and nutrition, and indicating the importance of intersectionality of gender with other social axes. This quote from a community health worker illustrates this point:

*“In my opinion, I think the only way proper child nutrition can be achieved is if the [young] wife and her husband are cooperative and work together. But the way things work here, where all the money [that is sent] must pass through the mother-in-law [senior woman in the home], then she decides how much she will give to her daughter-in-law, it can’t really work. Like if the young mother is given only 500 shillings [to last the whole month], how can she ensure proper nutrition for her child with that little money? It’s very hard…”* (CRF002, female community health worker)

In particular, the descriptions around *lugwizo* suggested the centrality of the mother in perceived illness causation and prevention. Unlike *kirwa,* where potential blame was apportioned equally to both men and women, for *lugwizo* the mother was described as a key determinant in whether or not the child acquired the illness. In fact one unique aspect of *lugwizo* was that it was sometimes defined as being dependent on the mother’s inherent attributes. That is, there were those mothers who were believed to innately have *lugwizo* which was either transmitted to their child through breastfeeding while pregnant, or predisposed their children to acquiring *lugwizo* through their blood. Then there were those mothers who could breastfeed while pregnant without adverse consequences for the child.

*“Many people say that the children can continue breastfeeding even when their mothers fall pregnant and yet do not get lugwizo…it depends from parent to parent. Some mothers have lugwizo and some do not have lugwizo; even if they continue breastfeeding while they are pregnant it does not affect the child, but for others…I think it depends on one’s blood…”* (Kadzo homestead_visit 2, index child’s mother)

Additionally, although not specifically examined in depth, it sometimes seemed that women were considered primarily responsible for ensuring appropriate child spacing, in order to avoid *lugwizo*. There was rarely spontaneous discussion amongst respondents of the role men played in women falling pregnant “too quickly”, or of women’s potential lack of negotiating power with regards to sexual relations within the home. Nevertheless, the data strongly suggest that women were generally held accountable for a child’s ill health if she fell pregnant “too soon” following her previous pregnancy. Moreover, it was mostly the women who blamed themselves or their fellow women for a child acquiring *lugwizo.*

*“Maybe I will speak for myself, like this child of mine, I was unfair to her. I fell pregnant too quickly after giving birth to her…I would just sit by myself crying and lamenting “why have I done this to my child, falling pregnant so quickly when she is still an infant?” (CRF003, female community health worker)*

The intersection between gender and age in relation to child health was also suggested in discussions regarding *kirwa*. Senior women were stated as specifically playing an important role both in the prevention and management of the problem. Though all respondents mentioned traditional healers as the means through which *kirwa* was treated, some made specific reference to elder or senior women as healers; or as those with easy access to the medicine required to either treat or avert *kirwa,* particularly in cases of suspected marital infidelity. These older women were described as giving certain traditional medicines to their daughters-in-law in advance of child birth to prevent the child from being born with *kirwa*.

*“A lot of the senior women have these medicines. That’s why sometimes, especially for the couples who live in the city, you’ll hear the mother-in-law [summon the daughter-in-law before she gives birth]. When the daughter-in-law goes to see her, she will inquire about the husband’s behaviour…Then the senior woman will say, “My daughter, I didn’t call you here with any ill will, but according to our Giriama culture, there is this medicine that a pregnant woman is given to take. Take it so that in case your husband went out of the marriage, it will save you both from embarrassment.””* (CRF001, female community health worker)

## Discussion

The findings from this qualitative study undertaken in Kilifi County, Kenya on the recognition of childhood undernutrition have shown that specific local taxonomies relating to undernutrition partially overlapped with biomedical classifications. The influence of biomedical concepts in local aetiologies of undernutrition and suggested treatment options was evident. However, local terminology (*kirwa*) was primarily employed where the causes were perceived to be the result of social deviance or supernatural influences. In such cases biomedical or naturalistic treatments were thought to be of little use.

Overlaps in biomedical and local taxonomies have been reported elsewhere. In a study of local understandings of malaria in south-eastern Tanzania [41] for example, the local model of malaria was primarily drawn from the biomedical model although the two were not entirely congruent. The biomedical model of malaria was sometimes complemented with a logic of witchcraft. This was especially the case when all the events happening during an actual illness episode could not be explained biomedically; for instance where illness persisted despite antimalarial treatment [41]. The authors suggested that the local and biomedical models were employed in two ways; either exclusively, where the two models co-existed and were used as alternative distinct explanations, or in a complementary fashion where the two models of illness causation merged and were perceived to act concurrently in explaining illness experience [41]. Notions of exclusivity and complementarity in illness causation did not emerge as strongly in our study of undernutrition. Nonetheless, despite the influence of the biomedical paradigm in the study community, these concepts might help explain the fluid boundaries associated with discussions of *kirwa*.

The three illnesses associated with child undernutrition *(*kwashiorkor*, kirwa* and *lugwizo),* were considered to be potentially serious and to have significant adverse consequences to children’s health and wellbeing unless specific attempts were made to treat the condition. By contrast, moderate acute malnutrition - the form targeted by the SFP - was minimally recognised in children. Low weight was clearly identified but it was not considered a significant health problem, rather a concern that was quite routine and manageable. This likely reflects low weight as being common in this community, resulting in normalization of the condition. Normalization of childhood undernutrition has been observed in other settings. Chary et al. found that stunting in children in rural Guatemala was perceived as being normal partly due to the high rates of stunting in the region [42]. Similarly, Amuyunzu-Nyamongo & Nyamongo found that mothers in an urban slum community of Kenya tended to classify childhood illness into various categories including ‘acute’ and ‘chronic’ [24]. Undernutrition along with other illnesses was categorized as “*chronic therefore not requiring immediate action*”. So whilst undernutrition was not necessarily normalized, there was no sense of urgency associated with managing the problem as it was viewed as enduring or long-lasting [24]. This normalized view of poor health related to undernutrition in turn impacted on management of the problem and related decision-making.

Gender was a key theme in the discussions of causation of childhood illness associated with undernutrition and the management of the problem. Mothers were perceived to have primary responsibility for ensuring that their children were of good health and nutritional status including through; providing nutritious foods for their children, guaranteeing sufficient and appropriate breastfeeding, and maintaining adequate child spacing. In some cases, mothers were viewed as being inherent carriers of illnesses such as *lugwizo,* which was then passed on to their vulnerable children. Mothers were implicitly viewed as having failed in their role when children failed to thrive. The only exception to this was in relation to *kirwa* where both parents were seen as potentially culpable. Even then, there was additional onus on pregnant mothers to for example consume specific herbal medicines pre-natally, to avoid adverse effects of any potential infidelity on the part of the child’s father. Maternal blame for poor child health has been observed in other settings in sub-Saharan Africa [28, 33], as well as in this community in relation to sickle cell disease [43]. The sickle cell study reported that women – particularly sisters-in-law – played a significant role in ascribing blame to fellow women, and that there was a fundamental tendency within the study community for mothers to be held accountable for negative events relating to their children [43]. Child health management decisions particularly where the health problem was perceived as non-serious, such as moderate acute malnutrition, were perceived to be the responsibility of the mother. This gendered division of roles and responsibilities with regards to child health and care, and particularly the notion of women as the primary guardians of children’s health and nutrition has been observed in many other settings [17, 18, 23, 33, 34]. While men are often considered the ‘owners’ of the children, responsibility for their day to day health and well-being lies with the children’s mothers [27, 33, 43].

### Limitations

One key criticism of qualitative case studies is that they are highly context-specific, which can make it difficult to generalize the research findings to other contexts. This study, for instance, was conducted in a small rural setting at the coast of Kenya. Even if the results are useful in informing future implementation of child nutrition interventions in this setting, the findings are not necessarily applicable to other contexts. The results might be applicable to settings with similar social and economic backgrounds, but caution would need to be exercised in transferring the findings of this work to other settings. Thus, in this work the approach to generalizability was theoretical. Findings and areas of interest have been related to published literature, and emerging themes likely to be relevant across a wide range of settings have been identified. Thus, although it is a local study, the implications go beyond Kilifi and Kenya.

## Conclusion

Perceptions of child illness and gendered roles within households have an important influence on recognition and management of undernutrition including engagement with interventions. The findings from this study show that in this rural area of coastal Kenya, moderate acute malnutrition was not recognised as a health problem that aroused concern and required remedial action. Mothers also appeared to be likely to bear the blame for their children’s poor health. We suggest that our findings raise key issues for the design and implementation of nutrition interventions to tackle this problem. Furthermore, since moderate undernutrition appears to have been ‘normalized’ in this community, engagement and education needs to be provided to community members of the potential associated longer-term health consequences. Without such information, improving the nutritional status of moderately undernourished children is unlikely to become a primary concern and will remain the responsibility of women who may have little power to effect change.

The WHO and UNICEF have published recommendations for the integrated management of malnutrition that have been incorporated into national guidelines [10]. These provide a framework for community education and engagement. The guidelines clearly outline a process of community mobilization and engagement that entails continual community sensitization, active case-finding and home follow-up visits. There is, however, no explicit recognition in the guidelines of the influence of perceptions of illness in management of the problem and the potential role of gender or gendered responsibilities in child health and nutrition within communities. The guidelines should also include a need to explore local concepts in explaining the importance of moderate malnutrition; and recognise that local belief systems and perceptions of illness aetiology play an important role in management of the problem. Our findings also suggest that the guidelines need to pay specific attention to gendered nuances, including that young mothers may already be receiving ‘community blame’ for the poor condition of their children, and so may need additional support themselves in helping to treat their children. However this needs further exploration.

The normalization of moderate undernutrition in this community also suggests an on-going and pervasive problem that goes beyond the immediate and perhaps even the underlying causes. This implies the need for interventions that address the basic causes of undernutrition in the area as a whole. Whilst interventions that target immediate and underlying causes of undernutrition are useful, broader structural factors including global and local inequities that contribute to the problem need to be concurrently addressed. This could potentially be achieved by drawing on multi-sectoral approaches and designing interventions that are both nutrition-specific and nutrition-sensitive [8, 44]. Nutrition-specific interventions address the immediate determinants of child nutritional status. On the other hand, nutrition sensitive interventions address other determinants of child under-nutrition and include for example: broader poverty reduction initiatives; school meals programmes to encourage school attendance; women’s empowerment programmes that aim to improve the overall status of women; and water and sanitation programmes [8, 44].

Adequately implementing existing guidelines on management of acute malnutrition while paying attention to context-specific beliefs around illness aetiology and gendered-nuances; as well as employing approaches that address broader structural drivers is likely to result in better health and nutritional outcomes for children.

## Abbreviations

HH, household; SFP, Supplementary Feeding Programme; UNICEF, United Nation’s Children Fund; WHO, World Health Organisation
